# Outcomes after first percutaneous coronary intervention for acute myocardial infarction according to patient funding source

**DOI:** 10.1186/1472-6963-14-405

**Published:** 2014-09-18

**Authors:** Pamela J Bradshaw, Shauna Trafalski, Joseph Hung, Tom G Briffa, Kristjana Einarsdóttir

**Affiliations:** School of Population Health, The University of Western Australia, 35 Stirling Highway, Perth, WA Australia; Centre for Health Services Research, School of Population Health, The University of Western Australia, 35 Stirling Highway, Perth, WA Australia; School of Medicine and Pharmacology, Sir Charles Gairdner Hospital, Hospital Avenue, Nedlands, WA Australia; Telethon Kids Institute, University of Western Australia, PO Box 855, 6872 West Perth, Western Australia Australia

**Keywords:** Health care funding, Myocardial infarction, Cardiac procedures

## Abstract

**Background:**

Disparities in the use of invasive coronary artery revascularisation procedures to manage acute myocardial infarction (AMI) have been found in several developed economies. Factors such as socio-economic status, income and funding source may influence the use of invasive procedures and have also been associated with ongoing care. The objectives of this study were to determine whether outcomes for patients at one and five years after AMI treated with first-ever percutaneous coronary intervention (PCI) were the same for public and privately funded patients.

**Methods:**

Retrospective, population-based cohort study using linked data to identify 30-day survivors of AMI treated with PCI in the index admission between 1995 and 2008 in Western Australian hospitals. The main outcome measures were admission for another PCI, re-AMI, and all-cause and cardiac mortality at one and five years.

**Results:**

At one year, private patients were at greater adjusted risk for another PCI (HR 1.62 [1.36 – 1.94]; p < 0.001) than public patients, and more likely to have an additional revascularisation procedure from 90 days to 5 years (HR 1.33 [1.11 – 1.58]; p < 0.001). They were at less risk for all-cause death within five years (HR 0.69 [0.62–0.91]; p = 0.01) with a trend to reduced risk for cardiac death and re-AMI.

**Conclusions:**

Treatment as a private patient for AMI with first PCI is associated with an increased likelihood of additional coronary revascularisation procedure within 12 months and to five years, and a reduced risk for all-cause mortality to 5 years. While additional procedures were not associated with poorer outcomes, there was no clear relationship between better outcomes and additional procedures. Other lifestyle and health care factors may contribute to the significant reduction in all-cause mortality and the trends to reduced hazard for AMI and cardiac death among private patients.

## Background

Studies from several developed countries, including those with universal health care systems, report access to cardiac services for the management of acute myocardial infarction (AMI) may be influenced by factors other than clinical need. Socio-economic status [1], income [2, 3] and funding source [4–6] have been found to be associated with disparities in the use of invasive procedures such as coronary angiography, percutaneous coronary intervention (PCI) and coronary artery bypass graft (CABG) surgery, and with mortality. Similar factors have been shown to influence care after AMI, both in use of preventive medications [7–9] and further revascularisation procedures [9–11], while under-insurance has been linked to long-term survival after AMI [12].

This population-based study aims to determine whether funding source is associated with patient outcomes after a first-ever PCI for AMI. We used data linkage to compare repeat revascularisation, re-AMI, and all-cause and cardiac mortality at one and five years for public and privately-funded patients admitted from 1995 to 2008.

## Methods

### Data source

This study uses linked data extracted from the Western Australia (WA) Data Linkage System by the Data Linkage Branch of the Health Department of Western Australia. Briefly, the Data Linkage System systematically links available administrative health data (including hospital admissions and discharges and deaths) for the state. Records are collated under individual unique identifiers. Computerised probabilistic matching of patient names and other identifiers is used, with poorly-matched or unmatched records reviewed by a Linkage Officer. Validation of the matching for the hospital morbidity data estimated invalid links (false positives) and missed links (false negatives) at 0.11% each [13]. Hospitalisation data were available from 1985, providing 10 years of look-back.

### Case ascertainment

Patient selection, the identification of comorbidities, cardiac-related diagnoses, procedures and cause of death were based on the World Health Organization (WHO) International Classification of Disease (ICD) versions 9, 9-CM (Clinically Modified) and 10-AM (Australian Modification).

The study cohort included all individuals aged 35 years and over who were admitted to WA hospitals between 1 January 1995 and 31 December 2008 for an AMI (ICD-9, -9-CM ‘410.’, ICD-10 ‘I21’) as a principal diagnosis, were treated during admission with a first-ever PCI, and who had not undergone any type of revascularisation procedure in the previous 10 years. Revascularisation procedures were identified by codes for PCI (ICD–9 '5-363'; -9CM '36.01, 36.02, 36.05-36.07; ICD-10 blocks 670, 671) and CABG (ICD-9 '5-361', ICD-9-CM '36.10-36.19', ICD-10 blocks '672-679') (ICD–9 '5-361-5.362', -9CM '36.01-36.09, 36.10-36.2', ICD-10 blocks '670 to 679'). Those admitted as an emergency (i.e. not an elective admission) were selected, and only those who survived 30 days from admission were included in the survival analyses. This was done to reduce the possibility of case selection bias as most cases, public and private, are initially admitted to an emergency department in a tertiary hospital. Selection of funding status usually occurs after the initial critical care period so early in-hospital deaths would be disproportionately classified as 'publicly-funded'.

### Outcomes measures and study variables

Outcomes were events within one year (cases to end 2007, n = 5451) and cumulative events of first additional PCI after the index procedure, first readmission for AMI, and all-cause and cardiac death to five years. One year outcomes were measured from 30-days, and five-year outcomes were measured from 90 days post-index admission to minimise counting re- admissions and procedures related to care for the acute episode. Coronary artery bypass grafting (CABG) was included in the additional revascularisation procedure to five years. Re-admissions could be either emergency (unplanned) or booked (planned) admissions.

Patients were grouped by decades of age (<50 years, 50-59, 60-69, 70-79 and 80 years and over).

The index admission funding source was used to categorise patients as public or private. Public patients were all those who elected treatment under the Australian Health Care Agreements and Reciprocal Health Care Agreements. Private patients included those funded through private health insurance or self-funded (regardless of whether treated in a public or private hospital). As Australians can move freely in and out of private insurance, with multiple changes in status possible before and after the index admission, funding source was not used as a time-dependent covariate.

The linked data included socio-economic and geographical data in the form of the Index of Relative Socio-Economic Disadvantage (IRSD) for the area in which the patient resides, and the Accessibility/Remoteness Index of Australia (ARIA+), which indicates the road distance from and access to major services. These data were collected in census years 1996, 2001 and 2006 [14–16]. The scores used were those released nearest (before or after) the date of the index admission. Percentiles of the IRSD were used to categorise the area of residence into quartiles of relative socio-economic disadvantage, with Q1 (highest scores) being most disadvantaged. ARIA + values of 1 or 2 (i.e. major city and inner regional) were categorised as ‘urban/regional’ and values of 3, 4 or 5 (i.e. outer regional, remote and very remote) as ‘rural/remote’ [17]. These data were not available for 11% of patients.

A history of prior AMI, heart failure (HF) (ICD-9/9-CM '428.' and ICD-10 'I50.') and the number of admission for ischemic heart disease (IHD) (ICD-9/9-CM '410.'-'414.' and ICD-10 'I20.'-'I25.') in the previous decade were identified from hospital morbidity data. IHD admissions were categorised as ‘none’ or ‘1 or more’. Re-AMI (non-fatal new AMI) was defined as re-admission with a principal diagnosis of AMI. Other comorbid conditions were identified and weighted to obtain a Charlson Comorbidity Index score for each patient, using methods developed for ICD-9 and ICD-10 [18, 19], excluding the condition responsible for hospital admission (AMI) and existing HF. The comorbidity scores were categorised as ‘0’, ‘1-2’, ‘3-5’ and ‘≥6’.

### Statistics

Descriptive statistics were used to examine the demographic and clinical characteristics of the cohort, with the χ2 tests of independence used to compare categorical variables for outcomes to one year, and the 't-test' to test the equality of mean age.

Kaplan-Meier (K-M) techniques were used to construct survival curves, with the log-rank test used to test differences in survival for each factor. Predictor variables significantly associated with the outcomes (all-cause and cardiac mortality, re-AMI, and readmission with an additional PCI to 1 year, and any additional revascularisation procedure to 5-years) were entered into the final multivariate models. After ensuring the assumptions of proportionality were met, Cox regression models (backwards selection) were used to identify the predictor variables associated with all-cause and cardiac mortality, re-AMI, and readmission with a PCI to 1 year and any revascularisation procedure to 5-years. Cases were censored at 31st December 2008. A two-sided ‘p’ value of 0.05 or less was considered statistically significant.

The data were analysed using SAS version 9.2 (SAS Institute Inc., Cary NC, USA) and IBM SPSS Statistics for Windows, Version 21.0. Armonk, NY: IBM Corp. statistical software.

### Ethics

The study uses de-identified linked administrative data for which we have received a waiver of informed consent under the provisions of section 2.3.6 of the Australian National Statement on Ethical Conduct in Human Research 2007.

The study was approved by the Human Research Ethics Committees at the University of Western Australia and Health Department of Western Australia.

## Results

During 1995-2008, 6,941 patients with AMI treated with an emergency (non-elective) first-ever PCI, and who survived for 30 days after admission to hospital, were identified. Of these, the 6,176 patients (89%) with complete data for all variables formed the study cohort; 4,450 (72%) being public patients. The demographic and clinical characteristics of the patients are shown in Table 1. It was the first cardiac admission within 10 years for 92% of cases. Of the publicly-funded patients 97% underwent their first PCI in a public hospital, while 66% of private patients were also treated initially in a public hospital. Of all PCIs performed, over 90% included stent insertion for both public and private patients. A greater proportion of private patients underwent another revascularisation procedure within 90 days (Table 2).

**Table 1:** **Demographic and clinical characteristics of 6, 176 public and private patients who survived 30 days from admission for AMI with first-ever PCI - 1995-2008**

	Patient funding source		
	Public n=4450	Public n=4450	
	n (%)	n (%)	p value
**Mean age (SD) years**	61.3 (12.6)	61.2 (10.8)	0.75
**Female**	1125 (25.3)	358 (20.7)	<0.001
**Indigenous**	171 (3.8)	4 (0.2)	-
Quartile of IRSD*			
**Q1**	1487 (33)	264 (15)	
**Q2**	1051 (24)	299 (17)	
**Q3**	1222 (28)	553 (32)	
**Q4**	690 (15)	610 (35)	<0.001
**Rural or remote residence**	414 (9.3)	126 (7.3)	0.01
**Previous IHD admission**	367 (8.2)	123 (7.1)	0.16
**History of AMI**	329 (7.4)	118 (6.8)	0.48
**History of heart failure**	86 (1.9)	24 (1.4)	0.16
**Heart failure (index admission)**	390 (8.8)	114 (6.6)	0.005
**Single-vessel PCI**	3948 (89)	1469 (85)	<0.001
**Charlson Comorbidity 'Score'**			
**0**	2038 (45.8)	914 (52.9)	
**1-2**	1366 (30.7)	521 (30.2)	
**3-5**	670 (15.1)	200 (11.6)	
**≥6**	376 (8.4)	91 (5.3)	<0.001

^*^Index of Relative Socio-economic Disadvantage - Q1 = greatest disadvantage.

**Table 2:** **Events and outcomes at 90 days, and one and five years after first PCI among 6,176 publicly- and privately-funded 30-days survivors of AMI**

	Patient funding source n (%)		p
	Public	Private	
**Within 90 days of index admission**			
Additional PCI in 30 days	88 (2)	50 (3)	0.01
Additional PCI or CABG in 90 days	275 (6)	190 (11)	<0.001
**12 months (from 30 days post admission) n = 5451**	n = 3961	n = 1490	
Additional PCI	329 (8)	193 (13)	<0.001
Re-AMI	158 (4)	65 (4)	0.29
Crude mortality (all-cause)	78 (2)	13 (1)	0.01
Cardiac death (% of all deaths)	42 (54)	4 (31)	-
**5 years (from 90 days post admission) n = 6148**	n = 4427	n = 1721	
Survival to additional revascularisation	0.91	0.90	0.004
Survival to re-AMI	0.94	0.96	0.003
Survival (all-cause death)	0.90	0.95	<0.001
Survival (cardiac deaths)	0.96	0.98	<0.01

Patients in the ‘most disadvantaged’ quartile had higher comorbidity, with a significant trend for the association between increasing disadvantage and increasing comorbidity (χ2 trend 66.4, p < 0.001).

### Study cohort

Compared to public patients a greater proportion of private patients were men, in quartile four of IRSD (least disadvantaged), and were in the lower categories of comorbidity. During the index admission a smaller proportion of private patients had a HF diagnosis recorded (indicating either a complication or a history of HF) while multi-vessel PCI was more frequent.

The 763 (11%) patients excluded for missing data did not differ in age, sex, proportion of private patients, or comorbidity. They were more likely to have had a previous admission for IHD (14% vs 8%, p < 0.001) and a history of AMI (14% vs 7% p < 0.001), but not HF. The KM survival estimates for both those with and without missing data were 0.98 (SE = 0.002, 0.004) at 12 months and 0.91 (SE = 0.006, 0.013) at 5 years.

### One year outcomes

There were 91 deaths within 12 months, of which half (46) were cardiac deaths. The outcomes at one year for publicly- and privately-funded patients are shown in Table 2. The hazard ratios (HR)s for public versus privately funded patients were obtained after adjustment for age-group, sex, HF, a history of AMI, prior admission for other IHD, Charlson Comorbidity Index category, quartile of IRSD, ARIA + (urban vs rural/remote) and single versus multi-vessel first PCI. An additional PCI within 30 days was also included in the models for re-AMI and survival to 1 year (Table 3).

**Table 3:** **Association of funding source with outcomes (adjusted hazard ratio) at one and five years after AMI with first-ever PCI**

Outcomes	Hazard ratio (95% CI)	p
	Private vs public funding	
**12 months (from 30 days post admission)**		
Additional PCI	1.62 (1.36-1.94)	<0.001
Re-AMI	1.37 (1.01-1.86)	0.04
All-cause mortality	0.61 (0.34-1.11)	NS
Cardiac death	0.42 (0.18-0.99)	0.05
**5 years (from 90 days post admission)**		
Additional PCI or CABG	1.33 (1.11-1.58)	0.002
Re-AMI	0.77 (0.59-1.01)	0.06
All-cause mortality	0.69 (0.52-0.91)	0.01
Cardiac death	0.63 (0.41-0.98)	0.04

Models adjusted for decade of age, sex, histories of AMI and HF, number of IHD admissions, HF in the index admission, Charlson Comorbidity Index score, single- or multi-vessel index PCI, Index of Relative Socio-economic Disadvantage (IRSD), ARIA + category and an additional revascularisation procedure or re-AMI in 90 days for 5-year outcomes.

PCI = percutaneous coronary intervention, AMI = acute myocardial infarction, CABG = coronary artery bypass graft.

#### Additional PCI

There were 164 additional PCI within 30-days of the index procedure among 30-day survivors of AMI (either during the index admission, which could include inter-hospital transfer, or in a re-admission) and 465 additional revascularisation procedures within 90 days. An additional PCI after 30 days and within one year was more frequent among privately-funded patients (Table 2). After adjustment for all clinical and demographic variables private patients were at an increased ‘hazard’ for an additional PCI within 12 months of the index AMI (Table 3), as were those with greater comorbidity.

#### Re-AMI

There were 223 patients re-admitted for AMI within the first year (Table 2). The association with funding source reached statistical significance, but with a broad confidence interval with the trend favouring privately-funded patients. There a significant interaction between funding source and IRSD (socioeconomic indicator). Being among the ‘least disadvantaged’ patients was associated with lower risk for re-AMI compared to the most disadvantaged. Greater comorbidity was associated with increased risk for re-AMI, as was HF recorded in the index admission.

#### Mortality

All-cause and cardiac deaths are shown in Table 2. Funding source was not associated with all-cause mortality to one year, but with a trend to a reduced hazard for cardiac death for privately-funded patients on low numbers (Table 3). Risk increased with older age, a history of HF, and increasing comorbidity.

### Five year outcomes

There were 29 deaths between 30 and 90 days of admission, leaving 6, 147 patients for follow-up to five years. Only11 re-AMIs were recorded between 30-90 days. Cumulative survival to each outcome to five years favoured privately-funded patients (Table 2).

#### Additional revascularisation procedure

Privately-funded patients were more likely to have a readmission with a revascularisation procedure within 5 years (Table 3). The ‘hazard’ for undergoing an additional revascularisation procedure decreased with increasing age while those with greater comorbidity were more likely to have an additional procedure as were those who underwent a multi-vessel PCI in the index admission.

#### Re-AMI

There was a trend to reduced risk for re-admission for AMI after 90 days among privately-funded patients although the confidence interval crossed unity (Table 3). Patients with HF in the index admission, those with greater comorbidity, and those who had undergone a multi-vessel index PCI were at increased risk for re-AMI, while patients aged between 50-80 years were at less risk for re-AMI than those aged <50 years; risk was non-significantly higher for those aged 80 years or more.

#### Survival

Survival to all-cause death and cardiac death at 5 years was poorer for publicly-funded patients (Table 2). The hazard for all-cause death at five years was reduced for those treated as private patients in the index admission (Table 3), with the effect of funding source seen across all age groups. Risk factors associated with an increased risk for death were age over 70 years, a history of heart failure and increasing comorbidity. Neither re-AMI nor an additional PCI in the 90 days after admission were associated with 5-year survival.

The reduction in hazard for a cardiac death for privately-funded patients was of statistical significance but the confidence interval was broad (Table 3). Older age and greater comorbidity were predictors for a cardiac death, and a history of HF more than quadrupled the hazard.

## Discussion

As expected, the factors most frequently associated with an increased risk for re-admission or death, older age, greater burden of comorbidity, HF and a history of IHD [20–22], were significant predictors of outcomes in this study. In addition, the source of patient funding in the index admission was associated with the likelihood of having an additional revascularisation procedure, and of reduced hazard of death to five years.

Those electing treatment as private patients, whether in a public or private hospital, were significantly more likely than public patients to undergo an additional procedure within 90 days and 12 months.

Explanations for the increased rates of additional procedures among private patients include a greater need for a repeat procedure to manage complications such as in-stent thrombosis, and more severe CAD. As cardiac deaths and readmissions for AMI were similarly low in the first year for both groups, and long-term survival was better for private patients, this explanation is unlikely.

As well as an additional PCI within the first three months after the index AMI, private patients were more also likely to have multi-vessel PCI during the index admission. While around 50% of ST-elevation AMI (STEMI) patients suffer multi-vessel disease, not all patients will undergo multi-vessel revascularisation, either at the index PCI or as a staged procedure [23]. While a staged strategy (treating non-culprit lesions in a planned intervention of additional PCI) is associated with lower risk for mortality than multi-vessel PCI during the acute management of AMI [23, 24] private patients appear to be in receipt of greater rates of both. Although these administrative data are insufficient to identify the number of diseased vessels at presentation, or the revascularisation strategy, the greater use of multi-vessel PCI during the index admission, and of additional PCI after discharge, suggests a greater focus on achieving complete revascularisation among privately-funded patients.

Less aggressive revascularisation among public patients may be to their disadvantage, as evidence from clinical trials and registries for patients with acute coronary syndromes or stable coronary disease treated with PCI suggests that incomplete revascularisation of multi-vessel disease is associated with a greater risk for major adverse coronary and cerebrovascular events and deaths in the longer term [24–26]. However, in our modelling, an additional revascularisation procedure within the first 30 to 90 days was not independently predictive of a re-AMI, or survival at 1 or 5 years; thus more aggressive interventional treatment did not explain the differences in long-term survival between private and public patients.

The greater use of additional procedures among private patients may also be related to the different level of access to PCI in the public and the private sectors. Patients treated for AMI in private hospitals in the Australian state of Victoria were found to be 2-3 times more likely to undergo angiography and PCI than public patients in public hospitals [6]. The authors concluded that fee-for-service payments might generate a financial incentive in the private system not present in the public system. This incentive could contribute to a tendency to re-admit private patients for an additional PCI, as seen in our study.

In a study of socio-economic inequalities and the use of coronary angiography and revascularisation procedures in Western Australia the 'more advantaged' patients admitted with angina, but not those with AMI, had higher rates of coronary procedures [27]. Similarly, in our cohort of patients with AMI, funding source, but not the Index of Relative Socio-economic Disadvantage, was associated with the use of procedures. The IRSD was independently associated only with risk for re-AMI within 12 months, with the ‘least disadvantaged’ only half as likely to suffer a re-AMI as those’ most disadvantaged’. This may reflect the overall poorer health status associated with socio-economic disadvantage, including greater comorbidity and a greater prevalence of risk factors [28, 29], or poorer access to, or uptake of, secondary prevention and cardiac rehabilitation after discharge [7, 30].

There was a reduced hazard for all-cause and cardiac death to 5 years among private patients, with a trend towards a reduced risk for re-AMI (although the p-value failed to reach statistical significance). While PCI has been associated with reductions of mortality and non-fatal AMI compared to optimal medical therapy in high-risk patients with non-STEMI and unstable angina to five years [31], this was not the case for stable angina [32] suggesting that an additional PCI, *per se,* is unlikely to improve survival. If the additional procedures among private patients in this study were in response to worsening coronary artery disease, we were unable to convincingly demonstrate a benefit of this strategy in reducing re-AMI, perhaps due to lack of power, as event numbers were low.

The reduced all-cause mortality at five years among those treated as private patients could not be attributed with certainty to a reduction in re-AMI or cardiac deaths, although there were trends to reduced hazards for cardiac death at one and five years and, less convincingly, re-AMI. Alternative explanations for the better overall survival among private patients include the overall better health of patients who can afford private care. The probability that public patients have poorer health is indicated by the greater proportion of public patients suffering HF and other comorbidities, but information on the severity of coronary disease and other risks is not available from the data. Greater clinical detail would help determine whether differences in the management of private and public patients are patient-related or practice-related.

Overall, greater use of an additional revascularisation procedure among the generally healthier private patients was to their apparent benefit, but the reductions in the long-term hazard for a re-AMI or cardiac death were of borderline statistical significance. Patients who choose (and can afford) private funding may also benefit from better care after discharge, leading to a reduction in risk for re-AMI in the longer term and to better survival, regardless of interventional management.

### Strengths

The strength of this study is the availability of 22 years of well-validated [33], routinely-collected and audited data for all inpatient episodes in all WA public and private hospitals.

Although 11% of the population cohort had missing socioeconomic status or residential locality data, there was no difference in survival for those excluded; the findings are therefore representative of outcomes for the WA population.

### Limitations

We lacked clinical detail on the severity of coronary artery disease and on the type of PCI (primary, rescue, facilitated or in response to angiography findings). In addition, ST-segment elevation AMI (STEMI) and non-STEMI are not consistently identified in ICD coding. There is no information on other risk factors, such as smoking behaviour, or on medications. All of these factors may influence outcomes.

## Conclusions

Admission as a private patient for an AMI treated with a first-ever PCI is associated with a greater likelihood of having an additional revascularisation procedure early or late after admission, and reduced risk for all-cause mortality to five years compared to public patients. There was no apparent risk for worse outcomes associated with the more aggressive interventional management among private patients, although additional procedures within 90 days of the index PCI did not predict reduced risk for all-cause or cardiac death, or re-AMI. The benefits among those who can afford private health care may be associated with more aggressive treatment, or with other factors related to their generally higher socio-economic status. It remains uncertain as to whether public patients, who have a greater burden of comorbidity and likely poorer overall health status, would benefit from more aggressive management of their coronary artery disease.

## Abbreviations

WA: Western Australia
ICD: International Classification of Disease
AMI: Acute myocardial infarction
PCI: Percutaneous coronary intervention
CABG: Coronary artery bypass grafting
IHD: Ischaemic heart disease
IRSD: Index of relative socio-economic disadvantage
STEMI: ST-elevation AMI
HF: Heart failure.
